# Determination of Trace Nickel in Water Samples by Graphite Furnace Atomic Absorption Spectrometry after Mixed Micelle-Mediated Cloud Point Extraction

**DOI:** 10.3390/molecules23102597

**Published:** 2018-10-10

**Authors:** Quan Han, Yanyan Huo, Longhu Yang, Xiaohui Yang, Yaping He, Jiangyan Wu

**Affiliations:** 1 School of Chemical Engineering, Xi’an University, Xi’an 710065, China; huoyeye@163.com (Y.H.); sheepyangxh@163.com (X.Y.); hejin04c@126.com (Y.H.); 2 School of Chemistry and Chemical Engineering, Yan’an University, Yan′an 716000, China; 15991518624@163.com (L.Y.); wgy143@163.com (J.W.)

**Keywords:** nickel, cloud point extraction, mixed micelle medium, graphite furnace atomic absorption spectrometry, water samples

## Abstract

A simple and sensitive cloud point extraction method for the preconcentration of ultra-trace amounts of nickel as a prior step to its determination by graphite furnace atomic absorption spectrometry was proposed. It is based on the reaction of nickel with 2-(5-bromo-2-pyridylazo)-5-dimethylaminoaniline (5-Br-PADMA) in HAc–NaAc buffer media and mixed micelle-mediated extraction of the complex using the anionic surfactant sodium dodecyl sulfate sodium (SDS) and non-ionic surfactant (1,1,3,3-Tetramethylbutyl)phenyl-polyethylene (Triton X-114). The optimal reaction and extraction conditions such as pH, concentration of 5-Br-PADMA, SDS and Triton X-114, equilibrium temperature, incubation, and centrifuge time were evaluated and optimized. Under the optimal conditions, the calibration graph was linear over the range 0.1–5.5 ng/mL of nickel with a correlation coefficient of 0.9942. The detection limit obtained was 0.031 ng/mL, and the relative standard deviation was 2.1% for nickel (*c* = 2 ng/mL, *n* = 6). The proposed method was successfully applied to the determination of nickel in water samples.

## 1. Introduction

Nickel is an essential trace element for human beings, animals, micro-organisms, and plants. Nickel has also been identified as a component in a number of enzymes, participating in important metabolic reactions. When too little or too much nickel is taken up, either deficiency or toxicity symptoms can occur [1]. Thus, the determination of trace nickel in environmental and biological samples is becoming increasingly important for public health and the environment. However, determination of trace nickel in environmental and biological samples is very difficult because of its very low concentration and the interfering effects of the matrix. A highly sensitive analytical technique combined with preconcentration and separation approach is often required.

Several analytical methods such as adsorptive cathodic stripping voltammetry (ACSV) [2,3], instrumental neutron activation analysis (INAA) [4,5], graphite furnace atomic absorption spectrometry (GFAAS) [6], inductively coupled plasma atomic emission spectrometry (ICP-AES) [7,8,9], and inductively coupled plasma mass spectrometry (ICP-MS) [10,11] have been developed for the determination of nickel at low concentrations. GFAAS was used here because it is a well-established, as well as cost-effective, technique with excellent sensitivity and low volumes of sample required, and the equipment is available in many laboratories.

A variety of procedures for preconcentration of nickel, such as dispersive liquid–liquid extraction (DLLME) [12,13], solid phase extraction (SPE) [14,15], membrane extraction [16], and cloud point extraction (CPE) [17,18], have been developed. Among them, CPE, based on surfactant-mediated phase separation, has become an attractive area for separation and preconcentration of trace metal ions in recent years. The main advantages of CPE are low cost, convenience and simplicity, high preconcentration factors, and environmental safety. Furthermore, CPE fits well to the principles of the “green chemistry”.

The combination of CPE with GFAAS can take their respective advantages. Sun, Z.M. et al. and Garcia, S. et al. combined CPE with GFAAS for the determination of nickel in environmental and biological sample using methyl-2-pyridylketone oxime (MPKO) and 1-phenyl-3-methyl-4-benzoyl-5-pyrazolone (PMBP), respectively, as chelating reagents [6,19]. Pyridylazo dyes are an important class of organic chelating reagents. Among them, O-amio pyridylazo compound has unique “N,N,N” coordination structure (all the coordinate atoms are nitrogen atom), which can only react with nitrophlic metal ions, and thus has excellent selectivity. In our previous studies, we have reported CPE methods for preconcentration of cobalt, palladium, and rhodium, using O-amio pyridylazo reagents [20,21,22,23]. The results of our previous studies show much promise of CPE methods using O-amio pyridylazo dyes as chelating agent for preconcentration of metals for their good selectivity and sensitivity. The goal of this work is to combine GFAAS, as a highly sensitive detection technique, with CPE, as a highly efficient separation and preconcentration approach, and to develop a simple and highly efficient method for the determination of trace nickel in water samples. In the developed system, a laboratory-synthesized O-amio pyridylazo reagent 2-(5-bromo-2-pyridylazo)-5-dimethylaminoaniline (5-Br-PADMA) was selected as the chelating agent. The color reaction of 5-Br-PADMA with Ni^2+^ has been reported in previous works [24]. It was shown that Ni^2+^ could react with 5-Br-PADMA in the presence of anionic surfactant sodium lauryl sulfate (SLS) to form a ternary complex. Nonionic surfactant Triton X-114 was employed as the extractant. When the system temperature is higher than the cloud point temperature (CPT) of the mixed surfactant of Triton X-114 and SLS, the nickel complex could be extracted into the surfactant-rich phase. The analyte in surfactant-rich phase was then determined by GFAAS. The preconcentration and combination parameters of CPE/GFAAS technique were investigated. The detection limit of the proposed method is much lower than the reported CPE-GFAAS methods [19,20]. The method was applied for the determination of nickel in water samples.

## 2. Experimental

### 2.1. Apparatus

All atomic absorption measurements were performed with a TAS-990 atomic absorption spectrophotometer (Beijing Purkinje General Instrument Co. Ltd, Beijing, China) with deuterium arc background correction and a GFH-990 graphite furnace atomizer system. A nickel hollow cathode lamp (Beijing Shu Guang-Ming Electronic Lighting Instrument Co., Ltd., Beijing, China) was used as the radiation source. Argon was used as the purge and protective gas. All measurements were performed using integrated absorbance (peak area). The optimum operating parameters for GFAAS are given in Table 1. pH measurements were performed by a PB-10 digital pH meter (Sartoris Scientific Instrument Co. Ltd, Beijing, China) supplied with a combined glass electrode. A HH-2 thermostatic bath (Kewei Yongxing Instrument Co. Ltd, Beijing, China), maintained at the desired temperature, was employed for cloud point temperature experiments and phase separation was assisted using an 800-1 centrifuge (Pudong Physical Instruments Factory, Shanghai, China).

### 2.2. Reagents and Solutions

All reagents were of analytical-reagent grade unless otherwise mentioned and ultrapure water (18.2 MΩ·cm) obtained from a Simplicity 185 (Millipore Company, Billerica, MA, USA) water purification system was used throughout. A standard stock solution of nickel (1000 µg/mL) was prepared by dissolving 1.000 g of purity nickel (99.9%) in 30 mL HNO_3_ (1:1), boiled gently to expel brown fumes, then diluting to 1000 mL with water. Working solutions were prepared by stepwise dilution of the stock solution with water. A 1.0 × 10^−3^ mol L^−1^ 5-Br-PADMA (laboratory-synthesized [25]) solution was prepared by dissolving 0.0320 g 5-Br-PADMA reagent in 1000 mL ethanol. Further, 0.5% (*m*/*v*) anionic surfactant SLS solution was obtained by dissolving 0.5 g SLS in 100 mL water. One percent (*m*/*v*) Triton X-114 (Sigma-Aldrich, Milwaukee, WI, USA) solution was prepared by dissolving 1.0 g Triton X-114 in 100 mL of the water. A buffer solution of pH 5.0 was prepared by adding 0.2 mol L^−1^ HAc slowly to a 0.2 mol L^−1^ NaAc solution to the required pH on the pH meter. The 0.1 mol L^−1^ HNO_3_-methanol solution was prepared by mixing of 0.2 mol L^−1^ HNO_3_ and methanol in equal volumes. All the water samples were provided by Xi′an Hydrographic Bureau, Xi′an, Shaanxi Province, China.

### 2.3. Extraction Procedure

For the formation of Ni^2+^-5-Br-PADMA-SLS ternary micellar complex, an aliquot of the nickel working standard or sample solution, a certain amount of buffer solution, 5-Br-PADMA ethanol solution, and SLS were placed in a 10 mL graduated conical centrifuge tube. The mixture was shaken well. Afterwards, for CPE, Triton X-114 solution was added, and diluted to 10 mL with ultrawater. The resultant solution was equilibrated at a temperature in a thermostated bath for certain time and then separation of the two phases was achieved by centrifugation. Upon cooling in an ice bath for 5 min, the surfactant-rich phase became viscous and retained at the bottom of the tube. Then, the bulk aqueous phase could be easily separated by simply inverting the tube.

### 2.4. Optimization Procedures

For the study of the effect of pH, transfer 3.0 mL of 10 ng/mL standard solution of nickel to each of eight 10 mL graduated conical centrifuge tubes, add 2 mL pH 3.5, 4.0, 4.5, 5.0, 5.5, 6.0, 7.0 and 8.0 HAc-NaAc bufer solution in order. Then, 0.25 mL of 5.0 × 10^−4^ mol L^−1^ 5-Br-PADMA ethanol solution, and 1.0 mL 0.5% SLS were added to each. The mixtures were shaken well. Afterwards, 0.8 mL of 1% (*m*/*v*) Triton X-114 solution was added to each, and diluted to 10 mL with ultrawater. Proceed as described in the optimized procedure below (Section 3.6).

For the investegation of effect of the amount of 5-Br-PADMA, 3.0 mL of 10 ng/mL standard solution of nickel and 2.0 mL pH 5.0 HAc-NaAc bufer solution was placed to each of nine 10 mL graduated conical centrifuge tubes. Add 0.08, 0.10, 0.15, 0.20, 0.25, 0.30, 0.35, 0.40 and 0.50 mL of 5.0 × 10^−4^ mol L^−1^ 5-Br-PADMA ethanol solution in order. Then, 1.0 mL 0.5% SLS was added to each. The mixtures were shaken well. Afterwards, 0.8 mL of 1% (*m*/*v*) Triton X-114 solution was added to each, and diluted to 10 mL with ultrawater. Proceed as described in the optimized procedure below (Section 3.6).

For the study of the effect of SLS, 3.0 mL of 10 ng/mL standard solution of nickel, 2 mL pH 5.0 HAc-NaAc bufer solution and 0.25 mL of 5.0 × 10^−4^ mol L^−1^ 5-Br-PADMA ethanol solution were placed to each of seven 10 mL graduated conical centrifuge tubes. Add 0.10, 0.30, 0.50, 0.70, 0.90, 1.10, and 1.40 mL 0.5% SLS in order. The mixtures were shaken well. Afterwards, 0.8 mL of 1% (*m*/*v*) Triton X-114 solution was added to each, and diluted to 10 mL with ultrawater. Proceed as described in the optimized procedure below (Section 3.6).

For the investegation of effect of the amount of Triton X-114, 3.0 mL of 10 ng/mL standard solution of nickel, 2.0 mL pH 5.0 HAc-NaAc bufer solution, 0.25 mL of 5.0 × 10^−4^ mol L^−1^ 5-Br-PADMA ethanol solution and 1.00 mL 0.5% SLS were placed to each of ten 10 mL graduated conical centrifuge tubes. The mixtures were shaken well. Afterwards, add 0.10, 0.20, 0.30, 0.40, 0.60, 0.80, 1.0, 1.2, 1.4 and 1.6 mL 1% (*m*/*v*) Triton X-114 solution in order, and diluted to 10 mL with ultrawater for each. Proceed as described in the optimized procedure below (Section 3.6).

For the investegation of effect of equilibrium temperature, 3.0 mL of 10 ng/mL standard solution of nickel, 2.0 mL pH 5.0 HAc-NaAc bufer solution, 0.25 mL of 5.0 × 10^−4^ mol L^−1^ 5-Br-PADMA ethanol solution and 1.00 mL 0.5% SLS were placed to each of nine 10 mL graduated conical centrifuge tubes. The mixtures were shaken well. Afterwards, add 1.0 mL 1% (*m*/*v*) Triton X-114 solution in order, and diluted to 10 mL with ultrawater for each. The resultant solutions were equilibrated at 40, 45, 50, 55, 60, 65, 70, 75 and 80 °C in a thermostated bath for 10 min in order. Proceed as described in the optimized procedure below (Section 3.6).

For the investegation of effect of equilibrium time, 3.0 mL of 10 ng/mL standard solution of nickel, 2 mL pH 5 HAc-NaAc bufer solution, 0.25 mL of 5.0 × 10^−4^ mol L^−1^ 5-Br-PADMA ethanol solution and 1.00 mL 0.5% SLS were placed to each of five 10 mL graduated conical centrifuge tubes. The mixture were shaken well. Afterwards, add 1.0 mL 1% (*m*/*v*) Triton X-114 solution in order, and diluted to 10 mL with ultrawater for each. The resultant solutions were equilibrated at 60 °C in a thermostated bath for 5, 10, 15, 20 and 30 min in order. Proceed as described in the optimized procedure below (Section 3.6).

For the study of the effect of foreign Ions, 3.0 mL of 10 ng/mL standard solution of nickel, 2 mL pH 5 HAc-NaAc bufer solution, was placed a 10 mL graduated conical centrifuge tube. Add different amount of a foreign ion. Then, proceed as described in the optimized procedure below (Section 3.6).

### 2.5. Quantification Procediure

For each water sample, 3 mL of water sample was taken in a 10 mL graduated conical centrifuge tube, and then proceed as described in the optimized procedure below (Section 3.6). The analytical results were obtained with the calibration curve method. The calibration curve was obtained as follows: Transfer 0, 0.1, 0.2, 0.5, 1.0, 1.5, 2.5, 3.5, 4.5, and 5.5 mL of 10 ng/mL standard solution of nickel to each of ten 10 mL graduated conical centrifuge tubes. Proceed as described in the optimized procedure below (Section 3.6).

## 3. Result and Discussion

### 3.1. Effect of pH

**Figure 1:**
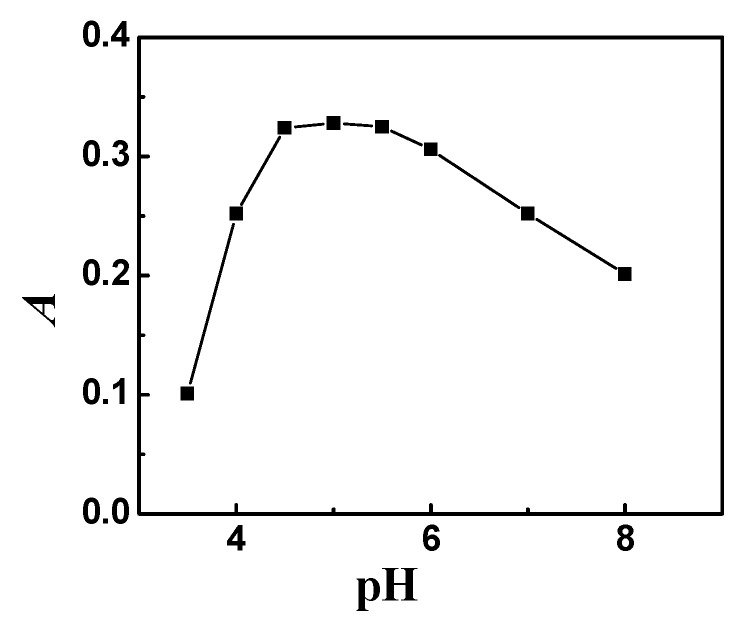
Effect of pH on absorbance (peak area). Thirty nanogram Ni; 0.25 mL 5 × 10^−4^ mol L^−1^ 2-(5-bromo-2-pyridylazo)-5-dimethylaminoaniline (5-Br-PADMA); 1.0 mL 0.5% (*m*/*v*) sodium lauryl sulfate (SLS); 0.80 mL 1.0 % (*m*/*v*) Triton X-114; temperature: 60 °C; heating time: 10 min.

The formation of metal chelate and its hydrophobicity were the two important factors that influenced CPE. The pH value played a unique role in metal chelate formation and subsequent extraction, and was proven to be one of the critical parameters for CPE. The effect of the pH on the extraction of nickel complex was studied in the pH range of 3.5–8.0. The results are shown in Figure 1. As shown, the optimum pH value is in the range of 4.8–5.3. Hence, pH of 5.0 was chosen for subsequent investigation.

### 3.2. Effect of the Amount of 5-Br-PADMA and SLS 

**Figure 2:**
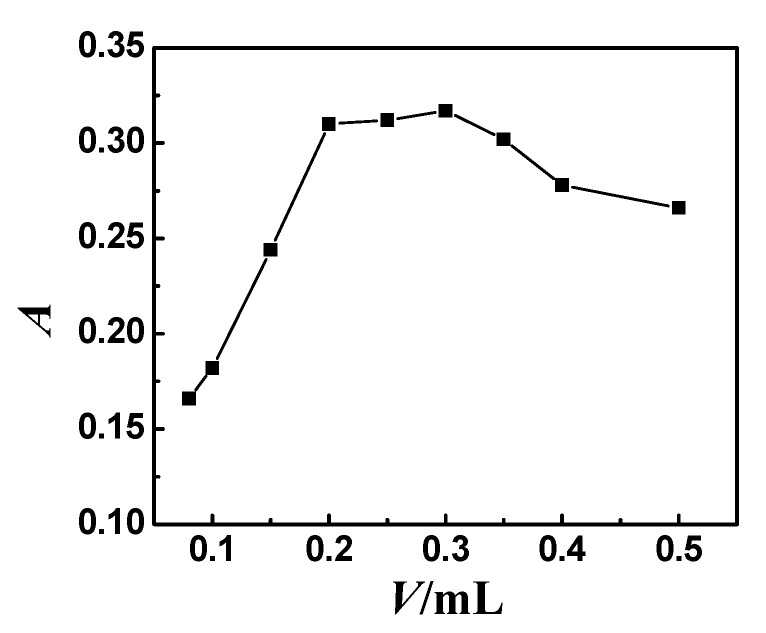
Effect of the amount of 5-Br-PADAM on absorbance (peak area). Thirty nanograms Ni; 1.0 mL 0.5% (*m*/*v*) SLS; 0.8 mL 1.0% (*m*/*v*) Triton X-114; temperature: 60 °C; heating time: 10 min; pH = 5.0.

As the chelating agent, 5-Br-PADMA was employed. The color reaction of 5-Br-PADMA with Ni^2+^ has been reported in previous works [23]. It was shown that Ni^2+^ could react with 5-Br-PADMA in the presence of anionic surfactant SLS to form a ternary complex. The effect of the amount of 5 × 10^−4^ mol L^−1^ 5-Br-PADMA on the CPE was investigated with the volumes of the 5-Br-PADMA from 0.08 to 0.5 mL. As shown in Figure 2, the absorbance (peak area) first increased and then remained stable in the presence of 0.20–0.30 mL 5.0 × 10^−4^ mol L^−1^ 5-Br-PADMA. When an excess amount of 5-Br-PADMA was used, a gradual decrease in the absorbance (peak area) was observed. As 5-Br-PADMA also has strong hydrophobicity, there was more 5-Br-PADMA and less Ni^2+^-5-Br-PADMA-SLS complex in the surfactant-rich phase with increasing amounts of 5-Br-PADMA. Hence, 0.25 mL of 5.0 × 10^−4^ mol L^−1^ 5-Br-PADMA was selected for subsequent experiments. The effect of the amount of 1% SLS on the absorbance (peak area) was also studied in the range of 0.1–1.4 mL. Maximum absorbance (peak area) value was achieved at 1.0 mL of 0.5% SLS. Therefore, 1.0 mL 0.5% SLS was chosen for the subsequent experiments.

### 3.3. Effect of the Amount of Triton X-114

**Figure 3:**
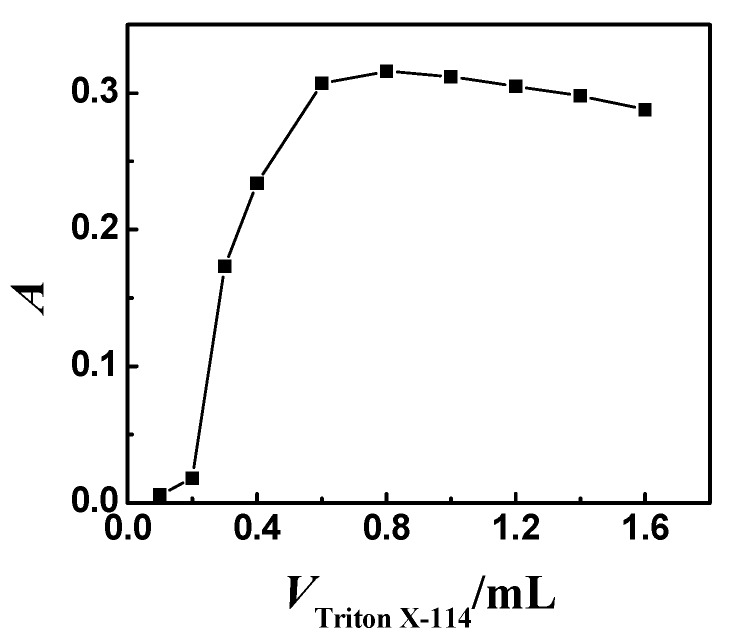
Effect of the amount of Triton X-114 on absorbance (peak area). Thirty nanograms Ni, 0.25 mL 5 × 10^−4^ mol L^−1^ 5-Br-PADMA; 1.0 mL 0.5% SLS; temperature: 60 °C; heating time: 10 min; 3.5 ng mL^−1^; pH = 5.0.

In the preliminary experiments, it was found that the addition of the nonionic surfactant, such as Triton X-114 to Ni^2+^-5-Br-PADMA-SLS ternary complex and heating, could make the solution turbid. This indicated that Ni^2+^-5-Br-PADMA-SLS ternary complex could be extracted by the CPE method. Hence, the effect of 1.0% (*m*/*v*) Triton X-114 on CPE was investigated. The results are shown in Figure 3. As demonstrated in Figure 3, the maximum absorbance (peak area) was obtained in the volume range of 0.6–1.2 mL. At lower concentrations, the absorbance (peak area) is low probably the result of the inability of the assemblies to entrap the ternary complex quantitatively. With an increase in Triton X-114 to above 1.2 mL, the absorbance (peak area) decreases because of the increasing volumes of the surfactant phase. Therefore, 0.8 mL 1.0% Triton X-114 was selected for subsequent experiments.

### 3.4. Effects of Equilibrium Temperature and Time

Equilibrium temperature and incubation time are two important and highly effective parameters in CPE. It was desirable to employ the lowest possible equilibrium temperature and the shortest equilibrium time, as a compromise between completion of extraction and efficient separation of phases. Thus, the effect of equilibrium temperature on CPE was investigated within the temperature range of 40–80 °C. It was found that the maximum analytical signal (absorbance) and good phase separation were achieved when the temperature was over 50 °C. Therefore, 60 °C was used as the equilibrium temperature. The effect of the equilibrium time on CPE was also studied within a range of 5–30 min by keeping the equilibrium temperature of 60 °C. It was also found that the analytical signal became almost constant after 10 min. Hence, an equilibrium time of 10 min was chosen as an optimum value.

### 3.5. Effects of Foreign Ions

The effect of various cations and anions on the preconcentration and determination of 3 ng/mL Ni was studied under the optimum conditions. The tolerance limit was defined as the amount of foreign ion causing a variation in the absorbance of the sample less than ±5%. The results are summarized in Table 2.

### 3.6. Optimized Procedure

Under the experimental conditions, the optimized procedure is as follows: First, a suitable amount of aqueous sample solution containing 1–5.5 ng of nickel was placed in a 10 mL graduated conical centrifuge tube. Then, 2 mL pH 5.0 HAc–NaAc bufer solution, 0.25 mL of 5.0 × 10^−4^ mol L^−1^ 5-Br-PADMA ethanol solution, and 1.0 mL 0.5% SLS were added. The mixture was shaken well. Afterwards, 0.8 mL of 1% (*m*/*v*) Triton X-114 solution was added, and diluted to 10 mL with ultrawater. The resultant solution was equilibrated at 60 °C in a thermostated bath for 10 min and then the two phases were separated by centrifugation for 15 min at 3500 rpm/min. After cooling in an ice bath for 5 min, the bulk aqueous phase was decanted by inverting the tube. The surfactant-rich phase in the tube was heated in water bath at 100 °C to remove the remaining water. The remaining surfactant-rich phase was dissolved in 50 µL 0.1 mol L^−1^ HNO_3_-methanol solution in order to reduce its viscosity. Of the diluted extract, 10 μL was injected directly into the furnace for determination of absorbance (peak area).

### 3.7. Calibration Curve, Precision, and Detection Limit

Under the optimized conditions for preconcentration of nickel, the calibration graph obtained optimization procedure was linear over a range of 0.1–5.5 ng/mL. The linear equation for nickel was *A* = 0.09857*c* + 0.06527, here *A* is the absorbance (peak area) and *c* is the nickel concentration in solution (ng/mL), and the correlation coefficient was *R* = 00.9942. The limit of detection for nickel, calculated according to three times the standard deviation of the blank signals (3*s*), was 0.031 ng/mL. The relative standard deviation (RSD) was 2.1% (for *c* = 2 ng/mL, *n* = 6). The preconcentration factor, calculated as the ratio of the aqueous solution volume (10 mL) to that of the surfactant rich phase volume after dilution with HNO_3_-methanol solution (50 µL), was 200.

Table 3 compares the characteristic data of the present method with those of reported methods using CPE prior to nickel determination. As seen from the table, the detection limit and preconcentration factor for method are comparable or better than those given by many methods in the table. In addition, enrichment factor can be improved by using larger sample volumes.

## 4. Application of Real Water Samples

The proposed method was applied to the determination of nickel in water samples by the calibration curve method. Recovery tests by adding different amount of nickel standard solution were also performed according to the same procedure, and the analytical results and the recovery are shown in Table 4. The recoveries are between 97.3% and 103.0%, indicating the accuracy and applicability of the proposed method for the determination of nickel in the real water samples.

## 5. Conclusions

In this study, we proposed a new and sensitive method for the determination of nickel by CPE combined with GFAAS using 5-Br-PADMA as chelating reagent and the nonionic surfactant of Triton X-114 as extractant. CPE is a simple, convenient, inexpensive, and environmentally-friendly method for preconcentration and separation of trace metals from aqueous solutions to very small volume of surfactant-rich phase. GFAAS is a well-established, as well as a cost-effective, technique with excellent sensitivity and low volumes of sample required. The combination of CPE with GFAAS can take their respective advantages. The developed method achieves satisfactory results when applied to the determination of nickel in real water samples. Because of the advantages mentioned above, the method can also be used for the determination of nickel in more complex samples such as plants and wastes.

## Figures and Tables

**Table 1:** Operating conditions for graphite furnace atomic absorption spectrometry (GFAAS).

Parameters	Value
Wavelength	232.0 nm
Slit	0.2 nm
Lamp current	4.0 mA
Filter coefficient	0.10
Pressure (Ar)	0.60 mPa
Injected volume	10.0 μL
Drying temperature	100 °C (Ramp 10 s, hold 15 s)
Ashing temperature	800 °C (Ramp 15 s, hold 15 s)
Atomization temperature	2000 °C (Ramp 0 s, hold 3 s)
Cleaning temperature	2100 °C (Ramp 1 s, hold 2 s)

**Table 2:** Effect of foreign ions on the preconcentration/determination of nickel.

Species	Foreign/Ni (*w*/*w*)	Species	Foreign/Ni (*w*/*w*)
Li^+^, K^+^, Mg^2+^, Ca^2+^, Sr^2+^, Zn^2+^, F^−^, Cl^−^, Br^−^, SO_4_^2−^	2500	Cu^2+^, Cr^3+^, Ir(IV), Mn^2+^	400
Ba^2+^, Pb^2+^, La^3+^	2000	Bi^3+^, Pd^2+^, Ce(IV)	200
Fe^3+^, Al^3+^	1000	Ag^+^, Pt(IV), Hg^2+^	100
Cd^2+^, As(V), Mo(IV), W(VI), Rh^3+^	500	Co^2+^	50

**Table 3:** Comparison of the proposed method with previously reported methods using cloud point extraction (CPE) prior to nickel determination. GFAAS—graphite furnace atomic absorption spectrometry; ICP—inductively coupled plasma; RSD—relative standard deviation.

Reagent *	Extractant	Detection System	PF/EF **	RSD%	LOD (ng∙mL^−1^)	Application	Ref.
DMG	Triton X-114	UV-vis	20/	1.04	4	Tap and river water	[26]
PAN	Triton X-114	UV-vis	5/	1.3	5	Green tea, coconut water	[27]
ACDA	Triton X-114	UV-vis	10/	-	10	Natural and waste water	[28]
8-HQ	Triton X-114	FAAS	50/61	2.18	0.52	Drinking and waste water	[29]
Br-PADAP	Triton X-114	FAAS	/74	4.7	0.2	Saline effluents	[17]
PAN	Triton X-114	FAAS	8/	1.8	2.4	River water	[18]
APDC	Triton X-114	FAAS	/46	-	0.52	Whole blood, serum	[30]
1-Nitroso-2-naphthol	PONPE 7.5	FAAS	40/29	2.89	1.09	Tap, river, sea, and treated waste water	[31]
Quinalizarin	Triton X-114	FAAS	/92	4.6	2.8	Tap and sea water	[32]
MPKO	Triton X-114	FAAS	30/58	-	2.1	Natural and wastewater, soil, blood	[33]
8-HQ	Triton X-114	ICP/OES	10/9.5	0.22–2.93	0.23	Produced water	[34]
PMBP	Triton X-100	GFAAS	/27	4.3	0.12	Water	[6]
DPKSH	Triton X-114	GFAAS	/27	-	0.14	Natural waters, urine, and honey	[19]
5-Br-PADMA	Triton X-114	GFAAS	200/	2.1	0.031	Well and river water	This work

* DMG: dimethylglyoxime; PAN: 1-(2-pyridylazo)-2-naphthol; ACDA: 2-amino-cyclopentene-1-dithiocarboxylic acid; 8-HQ: 8-hydroxyquinoline; Br-PADAP: 2-(5-bromo-2-pyridylazo)-5-diethilaminophenol; APDC: ammonium pyrrolidinedithiocarbamate; MPKO: methyl-2-pyridylketone oxime; PMBP: 1-phenyl-3-methyl-4-benzoyl-5-pyrazolone; DPKSH: di-2-pyridyl ketone salicyloylhydrazone. ** PF/EF: preconcentration factor/enrichment factor. OES: optical emission spectrometery.

**Table 4:** Determination results of nickel in the water samples.

Sample	Added (ng/mL)	Found * (ng/mL)	Recovery (%)
Well water	-	0.56 ± 0.01	-
	1.5	2.10 ± 0.06	103
	3.5	3.99 ± 0.01	98.0
River water 1	-	ND	-
	1.0	1.03 ± 0.04	103
	4.0	3.94 ± 0.011	98.4
River water 2	-	ND	-
	1.0	0.973 ± 0.026	97.3
	3.0	3.06 ± 0.07	102
River water 3	-	ND	-
	2.0	2.02 ± 0.07	101
	4.0	3.96 ± 0.09	99.0

* Mean of six experiments ± standard deviation. Well water: pH = 7.05, G = 1204 µS/cm, COD (chemical oxygen consumption) = 28.3, ρ(Cu, Zn) < 50 ng/mL, ρ(Se) < 0.3 ng/mL, ρ(As) < 0.2 ng/mL, ρ(Hg) < 0.01 ng/mL, ρ(Cd) < 0.1 ng/mL, ρ(Cr(IV)) < 4 ng/mL, ρ(Pb) < 1 ng/mL, ρ(Fe) < 30 ng/mL, ρ(Mn) < 10 ng/mL. River water 1: pH = 7.24, G = 376 µS/cm, COD = 24.4, ρ(Cu, Zn) < 50 ng/mL, ρ(Se) < 0.3 ng/mL, ρ(As) < 0.2 ng/mL, ρ(Hg) < 0.01 ng/mL, ρ(Cd) < 0.1 ng/mL, ρ(Cr(IV)) < 4 ng/mL, ρ(Pb) < 1 ng/mL, ρ(Fe) < 30 ng/mL ρ (Mn) < 10 ng/mL. River water 2: pH = 7.15, G = 2220 µS/cm, COD = 39.8, ρ(Cu, Zn) < 50 ng/mL, ρ(Se) < 0.3 ng/mL, ρ(As) < 0.2 ng/mL, ρ(Hg) < 0.01 ng/mL, ρ(Cd) < 0.1 ng/mL, ρ(Cr(IV)) = 4 ng/mL, ρ(Pb) < 1 ng/mL, ρ(Fe) < 30 ng/mL, ρ(Mn) < 10 ng/mL. River water 3: pH = 7.34, G = 1680 µS/cm, COD = 29.1, ρ(Cu, Zn) < 50 ng/mL, ρ(Se) < 0.3 ng/mL, ρ(As) < 0.2 ng/mL, ρ(Hg) < 0.01 ng/mL, ρ(Cd) = 0.1 ng/mL, ρ(Cr(IV)) = 4 ng/mL, ρ(Pb) < 1 ng/mL, ρ(Fe) < 30 ng/mL, ρ(Mn) < 10 ng/mL. ND: not detected. All the water samples and their analytical results were provided by Xi’an Hydrographic Bureau, Xi′an, Shaanxi Province, China.
